# Integration of High-Throughput Imaging and Multiparametric Metabolic Profiling Reveals a Mitochondrial Mechanism of Tenofovir Toxicity

**DOI:** 10.1093/function/zqac065

**Published:** 2022-12-24

**Authors:** Adam Pearson, Dominik Haenni, Jamal Bouitbir, Matthew Hunt, Brendan A I Payne, Ashwin Sachdeva, Rachel K Y Hung, Frank A Post, John Connolly, Stellor Nlandu-Khodo, Nevena Jankovic, Milica Bugarski, Andrew M Hall

**Affiliations:** Institute of Anatomy, University of Zurich, Winterthurerstrasse 190, CH-8057 Zurich, Switzerland; Center for Microscopy and Image Analysis, University of Zurich, Winterthurerstrasse 190, CH-8057 Zurich, Switzerland; Division of Molecular and Systems Toxicology, Department of Pharmaceutical Sciences, University of Basel, Klingelbergstrasse 50, CH-4056 Basel, Switzerland; Wellcome Centre for Mitochondrial Research, Newcastle University, Framlington Place, Newcastle upon Tyne, NE2 4HH, UK; Wellcome Centre for Mitochondrial Research, Newcastle University, Framlington Place, Newcastle upon Tyne, NE2 4HH, UK; Department of Infection and Tropical Medicine, Royal Victoria Infirmary, Queen Victoria Road, Newcastle upon Tyne NE1 4LP, UK; Genito-Urinary Cancer Research Group, Division of Cancer Sciences, University of Manchester, Manchester, M20 4GJ, UK; Department of Surgery, The Christie Hospital NHS Foundation Trust, 550 Wilmslow Road, Manchester M20 4BX, UK; King’s College Hospital and School of Immunology & Microbial Sciences, King’s College London, London, SE5 8AF, UK; King’s College Hospital and School of Immunology & Microbial Sciences, King’s College London, London, SE5 8AF, UK; UCL Centre for Nephrology, Royal Free Hospital, Rowland Hill Street, London NW3 2PF, UK; Institute of Physiology, University of Zurich, Winterthurerstrasse 190, CH-8057 Zurich, Switzerland; Institute of Anatomy, University of Zurich, Winterthurerstrasse 190, CH-8057 Zurich, Switzerland; Institute of Anatomy, University of Zurich, Winterthurerstrasse 190, CH-8057 Zurich, Switzerland; Institute of Anatomy, University of Zurich, Winterthurerstrasse 190, CH-8057 Zurich, Switzerland; Department of Nephrology, University Hospital Zurich, Rämistrasse 100, CH-8006 Zurich, Switzerland

**Keywords:** tenofovir, drug toxicity, kidney, mitochondria, imaging

## Abstract

Nephrotoxicity is a major cause of kidney disease and failure in drug development, but understanding of cellular mechanisms is limited, highlighting the need for better experimental models and methodological approaches. Most nephrotoxins damage the proximal tubule (PT), causing functional impairment of solute reabsorption and systemic metabolic complications. The antiviral drug tenofovir disoproxil fumarate (TDF) is an archetypal nephrotoxin, inducing mitochondrial abnormalities and urinary solute wasting, for reasons that were previously unclear. Here, we developed an automated, high-throughput imaging pipeline to screen the effects of TDF on solute transport and mitochondrial morphology in human-derived RPTEC/TERT1 cells, and leveraged this to generate realistic models of functional toxicity. By applying multiparametric metabolic profiling—including oxygen consumption measurements, metabolomics, and transcriptomics—we elucidated a highly robust molecular fingerprint of TDF exposure. Crucially, we identified that the active metabolite inhibits complex V (ATP synthase), and that TDF treatment causes rapid, dose-dependent loss of complex V activity and expression. Moreover, we found evidence of complex V suppression in kidney biopsies from humans with TDF toxicity. Thus, we demonstrate an effective and convenient experimental approach to screen for disease relevant functional defects in kidney cells in vitro, and reveal a new paradigm for understanding the pathogenesis of a substantial cause of nephrotoxicity.

## Graphical Abstract

**Graphical Abstract:**
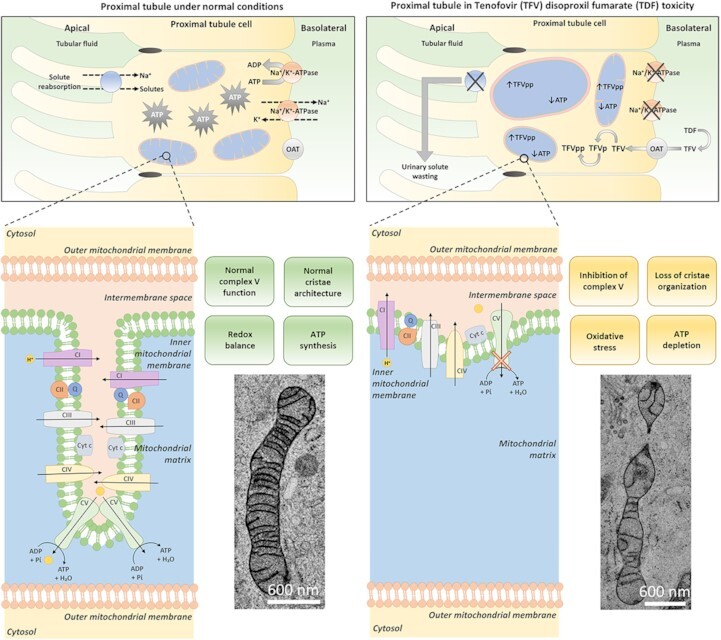

## Introduction

Toxicity from therapeutic drugs is a substantial cause of kidney disease, responsible for up to a quarter of cases of acute kidney injury (AKI),^1^ which occurs in 10%–15% of patients admitted to hospital, and over 50% in intensive care.^2^ Nephrotoxicity is also a leading reason for failure in drug development,^3^ accounting for ∼2% of attrition during preclinical studies and ∼20% in phase 3 studies.^4^ The proximal tubule (PT) is the most frequent site of damage, since it transports and metabolizes drugs, which can lead to intracellular accumulation of reactive metabolites.^5^ Proximal tubule cells are densely packed with mitochondria, which generate ATP to power reabsorption of filtered solutes. Mitochondria are often the victims of drug toxicity, and defects in mitochondrial function result in solute transport defects due to ATP depletion.^5^ However, in most cases, the mechanisms by which drugs damage mitochondria are not well understood, which limits options for intervention and prevention.

The predictive and mechanistic limitations of animal experiments have stimulated intensive efforts to generate better in-vitro cell models of nephrotoxicity, which can be subjected to detailed molecular screening.^6^ To derive meaningful information, it is critical that models display functional phenotypes relevant to patients, but the experimental conditions that produce these are often unknown. Importantly, differences in multiple variables—including baseline cell metabolism, antioxidant defenses, and drug uptake, accumulation, and processing—mean that concentrations required to reproduce patient phenotypes in vitro might be very different from typical plasma values in vivo. Therefore, high-throughput screening methods are needed to establish these models. High-content imaging is one such screening approach that has shown much promise,^7^ but methods to rapidly detect and quantify solute transport defects were previously lacking.

Tenofovir disoproxil fumarate (TDF) is a nucleotide reverse-transcriptase inhibitor (NtRTI) and archetypal nephrotoxin. It is a first line therapy for both HIV and Hepatitis B infections, and is also approved for prevention of HIV acquisition. Tenofovir disoproxil fumarate is an orally bioavailable prodrug that is rapidly metabolized to the active component tenofovir (TFV) in plasma. Once within cells, TFV is phosphorylated to TFV phosphate (TFVp), and then to TFV diphosphate (TFVpp), a structural analog of deoxyadenosine 5’-triphosphate (dATP) that inhibits elongation of viral DNA.^8^

Clinical studies have shown that a substantial proportion of TDF-treated individuals—more than 20% in cross-sectional studies,^9–12^ including in HIV uninfected^13^—display defects in PT transport function, with a spectrum of severity. Urinary wasting of substances normally reclaimed in the PT, such as phosphate, can lead to systemic depletion (renal Fanconi syndrome) and complications like bone mineral density loss and fractures.^14^ Moreover, TDF toxicity is associated with progressive loss of renal excretory function.^15^ A characteristic feature in biopsy specimens is the presence of enlarged, dysmorphic mitochondria, with striking abnormalities in cristae formation,^14,16^ strongly hinting that TDF is harmful to these organelles. These findings have been replicated in studies with rodent models.^17,18^ Meanwhile, both cell^19^ and animal experiments^20^ have shown that TDF induces oxidative stress. However, a mechanistic explanation for these well-recognized phenomena has remained elusive.

Older antiviral drugs were thought to induce mitochondrial defects by inhibition of mitochondrial DNA (mtDNA) polymerase gamma (POLγ) and depletion of mtDNA.^21^ It has therefore been postulated that TDF might exert a similar effect^22^; but numerous studies have failed to demonstrate a reduction in mtDNA abundance in cells,^23^ animals,^24^ or human biopsies,^25^ highlighting a clear need to explore other explanations. Experimentally, identifying disease relevant effects of TDF/TFV in cell models is complicated by the need to recreate the metabolic conversion to active reagents. Moreover, another substantial challenge is disentangling initial, drug-specific effects on mitochondrial function from generic secondary responses to metabolic stress. Finally, clinical studies show that TFV toxicity is a cumulative phenomenon,^26^ likely due to intracellular accumulation of harmful metabolites, further underlining that direct extrapolation of patient blood concentrations to in-vitro studies is overly simplistic.

Here, to investigate the metabolic phenotype of TDF toxicity, we have used a well-differentiated, human-derived cell line (RPTEC/TERT1), which replicates many of the major characteristics of PT cells in vivo.^27^ We designed a high-throughput imaging approach to generate quantitative readouts of solute transport and mitochondrial morphology, and leveraged this to screen the functional effects of TDF. Importantly, we confirmed that TDF was appropriately metabolized within these cells to generate the active antiviral metabolite TFVpp. From this, we were able to derive treatment protocols that reliably reproduce well-described features in patients.

By subjecting these in-vitro models to multiparametric metabolic profiling—including metabolomic screening, oxygen consumption measurements, and RNA-sequencing (RNA-Seq)—and integrating the results from each, we elucidated a highly robust molecular fingerprint of TDF toxicity. Critically, we identified that the active metabolite TFVpp inhibits complex V (ATP synthase), and that TDF treatment causes a rapid and dose-dependent decrease in complex V activity and expression, at a time point when other mitochondrial functions and pathways are well preserved, suggesting a specific effect. Because complex V is normally responsible for maintaining cristae formation and redox state, and generating ATP to power solute transport, this potentially explains the observed phenotype in humans.

## Methods

### Cell Culture and Reagents

RPTEC/TERT1 cells (Evercyte GmbH) were grown in ProxUp Basal Medium (excluding antibiotics) at 37°C. For all experiments, cells grew to confluency over 4–5 d, and then differentiated for 10 d before starting a treatment protocol. Cells form domes during the differentiation period. Chemicals were purchased from Sigma-Aldrich unless stated otherwise.

### Immunofluorescence Microscopy

Cells were grown in 96-well plates (655 090, Greiner) and washed with PBS, before being fixed with 3% PFA. Cells were permeabilized with 0.1% TritonX-100 and blocked for 30 min with 10% donkey and/or goat serum prepared in 1% BSA in PBS, and incubated with primary antibodies overnight at 4°C. Cells were incubated for 2 h with appropriate secondary antibodies (listed in the Supplementary Methods). To label nuclei, cells were incubated for 20 min with Hoechst (H1399, Molecular Probes). Cells were imaged using a fully automated high-content imaging system (IN Cell Analyzer 2500 HS, GE).

### Cellular Respiration

Cells were grown in 8-well culture miniplates (Seahorse XFp 103025-100, Agilent, Santa Clara, CA). The Cell Mito Stress Test (103 010–100, Agilent) was used to assess respiration. Seahorse XFp sensor cartridge ports were loaded with assay drugs dissolved in Seahorse base medium (103 334–100, Agilent) supplemented with 5 m m glucose, 1 m m pyruvate, and 4 m m glutamine. Oligomycin (1 µm), FCCP (1 µm), and Rotenone/Antimycin A (0.5 µm) were used to modulate the respiratory chain (RC). After injection of each drug, OCR was measured in 3 cycles, each consisting of 3 min of mixing followed by 3 min of measurement. Confluent cell monolayers were undisturbed following completion of treatment TDF protocols. Thus, upon commencing the assay, numbers of cells in treated and untreated groups were all but identical.

### Cellular ATP Concentration

Following treatment, cells were lysed on ice for 5 min using CelLytic M, and centrifuged to collect the supernatant, from which ATP was quantified using a bioluminescence assay (A22066, Molecular Probes). Luminescence was measured using a microplate reader (Synergy 2, BioTek). Protein concentration was determined using the QuickStart Bradford Protein Assay (5 000 006, Bio-Rad). Intracellular ATP concentrations were normalized to protein content.

### Quantifying the Relative Abundance of mtDNA to Nuclear DNA

Following treatment, DNA was extracted from cells using the DNeasy Blood and Tissue Kit (69 504, Qiagen). A 5ng aliquot of cellular DNA was used for each real-time quantitative PCR (qPCR). The abundance of mtDNA and nDNA was determined using a CFX96 real-time PCR detection system (Bio-Rad). Measurements were performed in duplicate with 100 n m of both sense and antisense primers in a final volume of 20 µL, using iQTM SYBR Green Supermix (1 708 880, Bio-Rad). Primers and PCR conditions are listed in Table S2A. PCR products were sequenced with the BigDye terminator kit (PerkinElmer Applied Biosystems), using an ABI3100 capillary sequencer (PerkinElmer Applied Biosystems). Relative abundance of mitochondrial genes over ACTB was calculated using the 2^−ΔΔCT^ formula.

### Assessment of ATP Synthase (Complex V) and Complex I Activity in RPTEC/TERT1 Cells

Cells were treated with 300 or 500 µm TDF for 24 h, before being permeabilized with digitonin (10 μg/mL). Alternatively, cells were permeabilized and then incubated with 500 µm TFVpp (NU-975, Jena Bioscience) for 30 min. Two million cells were suspended in MiR05 buffer. Complex V activity was measured using an Oxygraph-2k high-resolution respirometer equipped with DataLab software (Oroboros instruments), as described previously.^28^ To assess complex I activity, OCR was measured in permeabilized cells in the presence of the NADH-linked substrates glutamate (10 m m) and malate (2 m m).^29^

### ATP Synthase (Complex V) Activity Assay

The MitoTox Complex V OXPHOS Activity Assay Kit (ab109907, Abcam) was used. Tenofovir, TFVpp, and Oligomycin (103 010–100, Agilent) were dissolved in sterile water. Complex V activity was measured by monitoring change in absorbance at 340 nm.

### Electron Microscopy

Cells were grown on 6 mm sapphire disks (100 µm thickness, Engineering Office M. Wohlwend, Switzerland) coated with poly-l-lysine and gold-sputtered fiducial marks, and underwent high-pressure freezing, processing, and embedding, as previously described.^30^ Samples were imaged using a Talos 120 transmission electron microscope at 120 kV acceleration voltage equipped with a bottom mounted Ceta camera using Maps software (ThermoFisher).

### Metabolomics Analysis: Liquid Chromatography–Mass Spectrometry

Cells were washed twice with cold ammonium bicarbonate buffer before being detached by scraping in cold 80% methanol. Samples were transferred to a microcentrifuge tube and homogenized, before being centrifuged at 13 000 *g* for 3 min at 4°C. The supernatant was collected for analysis. A volume of 50 µL of methanol extract was dried under a nitrogen stream and reconstituted in 20 µL of mass spectrometry (MS) grade water, before being diluted with 80 µL injection buffer (composed of 90% acetonitrile, 9% methanol and 1% 5 M ammonium acetate). The dilution was vortexed and centrifuged at 16 000 *g* for 15 min at 4°C. A volume of 50 µL of the supernatant was transferred to a glass vial (Total Recovery Vials, Waters). Metabolites were separated on a nanoAcquity UPLC—equipped with a BEH Amide capillary column—coupled to Synapt G2Si mass spectrometer by a nano-electrospray source (Waters). MS1 (molecular ion) and MS2 (fragment) data were acquired using negative polarization and MS^E^ mode over a mass range of 50 to 1200 m/z at MS1 and MS2 resolution of >20 000.

MetaboAnalyst (version 4.0) was used to perform statistical analysis and unbiased MS peaks to pathways analysis. The Mummichog algorithm was used alongside a *P*-value cut-off, which captured the top 10% peaks. A mass accuracy tolerance of 3 ppm and the Homo sapiens MFN pathway library were used to generate a pathway activity profile. In addition, data were analyzed and detected ions were assigned identities using Progenesis QI software (Nonlinear Dynamics). Target metabolites were quantified by the area under the peak of the MS1 extracted ion chromatogram of the respective [M–H]^−^ ion (TFV: 286.0711 m/z, TFVp: 366.0374 m/z, TFVpp: 446.0037 m/z) using QuanLynx (MassLynx version 4.2, Waters).

### Transcriptomics Analysis: RNA-Seq

Total RNA extraction was performed using the RNeasy Mini Kit (74 104, Qiagen) and QIAshredder homogenizers (79 654, Qiagen). RNA quality was determined using a Qubit (1.0) Fluorometer (Life Technologies) and a Bioanalyzer 2100 (Agilent). Samples were processed using the TruSeq RNA Sample Prep Kit v2 (Illumina). The TruSeq SR Cluster Kit (Illumina) was used for cluster generation using 10 p m of pooled normalizer libraries. Sequencing was performed on a NovaSeq 6000 system (Illumina) with single end 100 bp using the TruSeq SBS Kit (Illumina).

Reads were quality-checked and aligned to the reference genome of Homo sapiens (GENCODE, GRCh38, release 32) with STAR version 2.7.3a.^31^ Distribution of the reads across genomic isoform expression was quantified using the R package GenomicRanges from Bioconductor Version 3.11. Differentially expressed genes were identified using the R package “edgeR”^32^ from Bioconductor version 3.11. Differentially expressed genes were used as an input for Gene Ontology-based pathway analysis using the R package “goseq.”^33^

### Gene-Metabolite Interaction Network Analysis

MetaboAnalyst (version 5.0) was used to perform gene-metabolite interaction network analysis on differentially expressed (*P* < .01) metabolites and genes—identified by metabolomics and transcriptomics. The network was refined by reducing the number of less important genes and metabolites by applying a degree filter of 5.

### Western Blot Analysis of RC Complex Abundance

Cells were lysed in the presence of a protease inhibitor cocktail (Roche) and homogenized. Mitochondrial and cytosolic cell fractionation was performed using a mitochondria isolation kit for cultured mammalian cells (89 874, ThermoFisher). Fractions were isolated by differential centrifugation and mitochondria pellets were resuspended in 2% CHAPS in TBS with protease inhibitors. Mitochondrial fractions were vortexed, clarified by centrifugation, and quantified using the BCA protein assay. Proteins were separated by SDS-PAGE and transferred on nitrocellulose membranes. Membranes were incubated with primary and secondary antibodies listed in Table S3. Signals were revealed with chemiluminescent HRP substrates (NEL113001EA, PerkinElmer Applied Biosystems) and bands were quantified using ImageJ.

### Immunofluorescence Staining for RC Complex Expression in Human Biopsies

Percutaneous renal biopsies were taken from people living with HIV and TDF-associated nephrotoxicity (*n* = 6). Open renal biopsies of nonpathogenic tissue adjacent to renal tumors were taken from HIV-uninfected individuals (*n* = 5). All biopsies were formalin-fixed and paraffin-embedded.

Two novel multiplex immunofluorescence assays^34^ were used to quantify mitochondrial mass and RC expression. Serial 4 µm sections were air dried, deparaffinized in Histoclear, and washed in water. Sections were rehydrated in a graded ethanol (EtOH) series (10 min 100% EtOH, 5 min 95% EtOH, and 5 min 70% EtOH). Antigen retrieval was performed with 1 m m EDTA pH 8.0 buffer for 40 min. Sections were incubated in 10% goat serum for 1 h and were then covered in a primary antibody cocktail for either complex I/IV or III/V (Table S4A), in a humidified chamber overnight. Following washes, sections were incubated in a secondary antibody cocktail (Table S4B) for 2 h. Sections were mounted in ProLong Gold Antifade Mountant (ThermoFisher) and imaged with a Zeiss Axio Imager M1 and Zen 2011 (blue edition) software, with a Monochrome Digital Camera (AxioCam MRm). Fourty proximal tubules were randomly identified per subject where available. Raw intensity values were corrected for background signal by subtracting the mean optical density from a no-primary control for each fluorophore. Values were normalized to VDAC1 intensity and expressed as Z-scores relative to the control biopsies.

### Statistics

Data are presented as mean ± SEM. Unpaired two-tailed *t*-tests were performed for comparisons between two groups; one-way ANOVA followed by Tukey’s or Dunnett’s multiple comparisons test for three or more groups. The strength of statistical significance is indicated by asterixis; *P*-values are indicated in the figure legends (values < .05 were considered statistically significant), alongside *n*-values and the statistical tests, which were executed. GraphPad Prism (version 8.4.3) was used for all statistical analysis.

### Study Approval

Human kidney biopsy samples were obtained for research purposes from residual diagnostic material under research ethics committee approval (17/NE/0015).

## Results

### Establishment of a High-Throughput Imaging Pipeline to Assess Functional Effects of TFV

**Figure 1.**
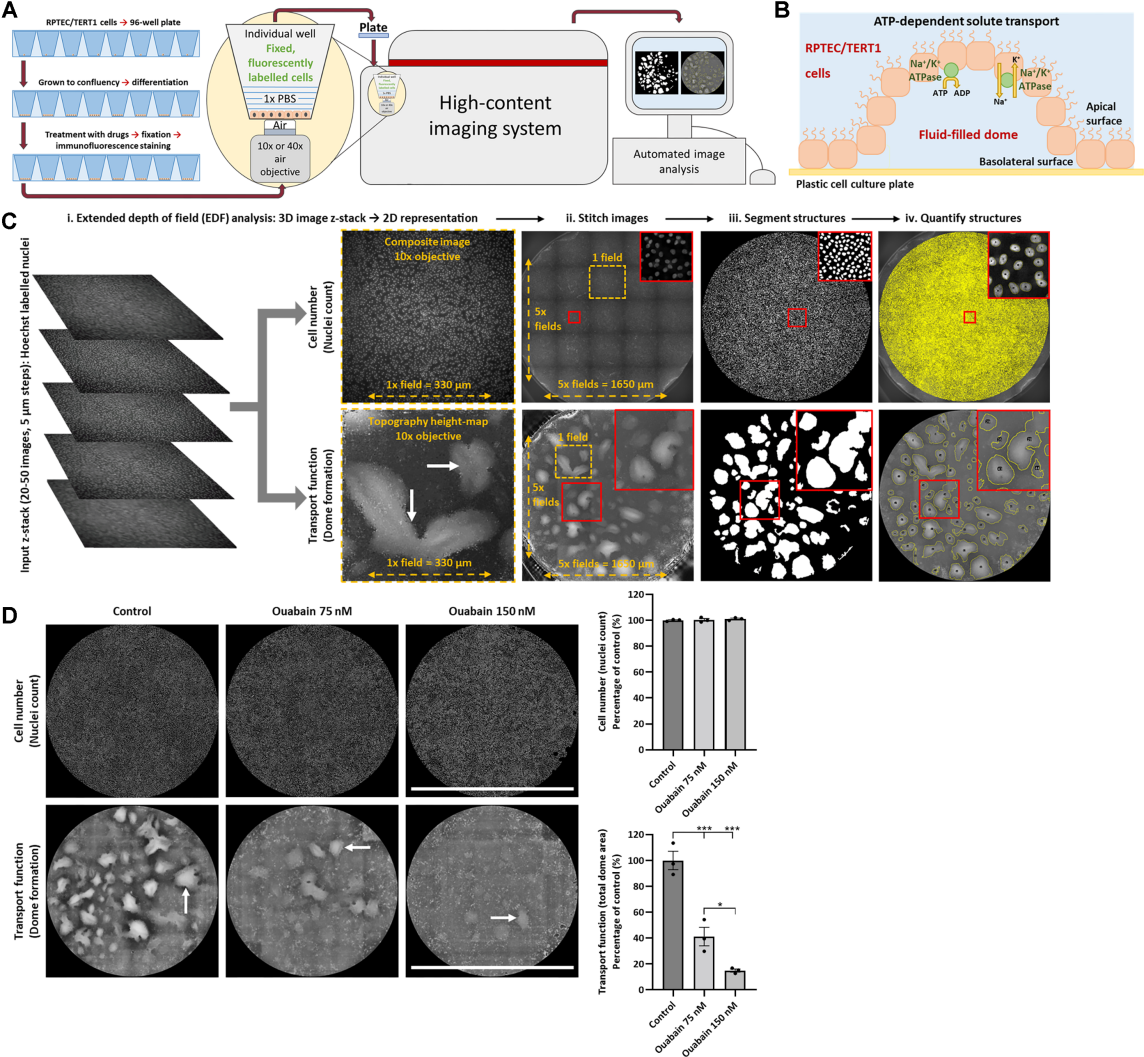
High-throughput imaging of solute transport function in human-derived proximal tubular cells.**(A)** Schematic of the imaging pipeline designed to assess dome formation in monolayers of differentiated RPTEC/TERT1 cells. **(B)** Basolateral sodium–potassium pumps (Na^+^/K^+^-ATPase) generate ion gradients that provide the driving force for proximal tubule (PT) transport processes. Domes occur due to fluid accumulation between the cell monolayer and the culture dish, driven by the activity of the Na^+^/K^+^-ATPase. Visible domes are preserved after fixation and provide a quantifiable readout of solute transport. **(C)** Extended depth of field (EDF) analysis was used to merge a z-stack of images into a single-focused composite image and a topography height-map of in-focus pixel positions. This allows 3D structures, such as domes, to be represented as a single in-focus 2D image. Twenty-five composite images and height-maps were stitched to recreate an entire cell monolayer consisting of approximately 75 000 cells. Machine learning was used to automatically identify, segment, and quantify structures of interest. Cell number was assessed by counting nuclei, whilst transport function was measured by calculating the total area of dome formation. Cells forming domes (white arrows) exist on a higher plane than surrounding cells and are identified in the height-map as a lighter color than cells attached to the cell culture plate. **(D)** Twenty-four-hours treatment with Ouabain—a Na⁺/K⁺-ATPase inhibitor—reduced dome formation (white arrows) without affecting cell number, demonstrating the physiological validity of our experimental workflow as a method to assess ATP-dependent PT transport function. Scale bar = 1650 µm. (*n* = 3, mean ± SEM, *P < .05, ***P < .001, one-way ANOVA with Tukey’s multiple comparisons test).

Since defects in solute reabsorption are the major clinical manifestation of nephrotoxicity in the PT,^5^ we developed a high-throughput imaging pipeline to measure solute transport in RPTEC/TERT1 cells (Figure 1A). Movement of filtered solutes from the apical to basolateral side of PT cells is driven by the Na^+^/K^+^-ATPase—using ATP generated by the RC—and results in the formation of fluid-filled domes when grown on an impermeable surface^35^ (Figure 1B). We therefore designed an algorithm to quantify total dome area in images acquired by an automated microscope (Supplementary Methods 1 and 2).

Z-stacks of 20–50 images were acquired in 5 µm steps. Extended depth of field (EDF) analysis was used to merge each stack into a single-focused composite image and a topography height-map of in-focus pixel positions to reveal the 3D structure of domes (Figure 1C). Composite images and height-maps were then stitched together to recreate an entire cell monolayer, typically consisting of around 75 000 cells. A machine learning-based approach was constructed to identify, segment, and quantify structures of interest within monolayers. Cell number was assessed by counting nuclei, whilst total dome area was used as a readout of transport function (Figure 1C). To validate this approach, cells were treated for 24 h with ouabain, an inhibitor of Na^+^/K^+^-ATPase, which produced a dose-dependent decrease in transport function (Figure 1D).

**Figure 2.**
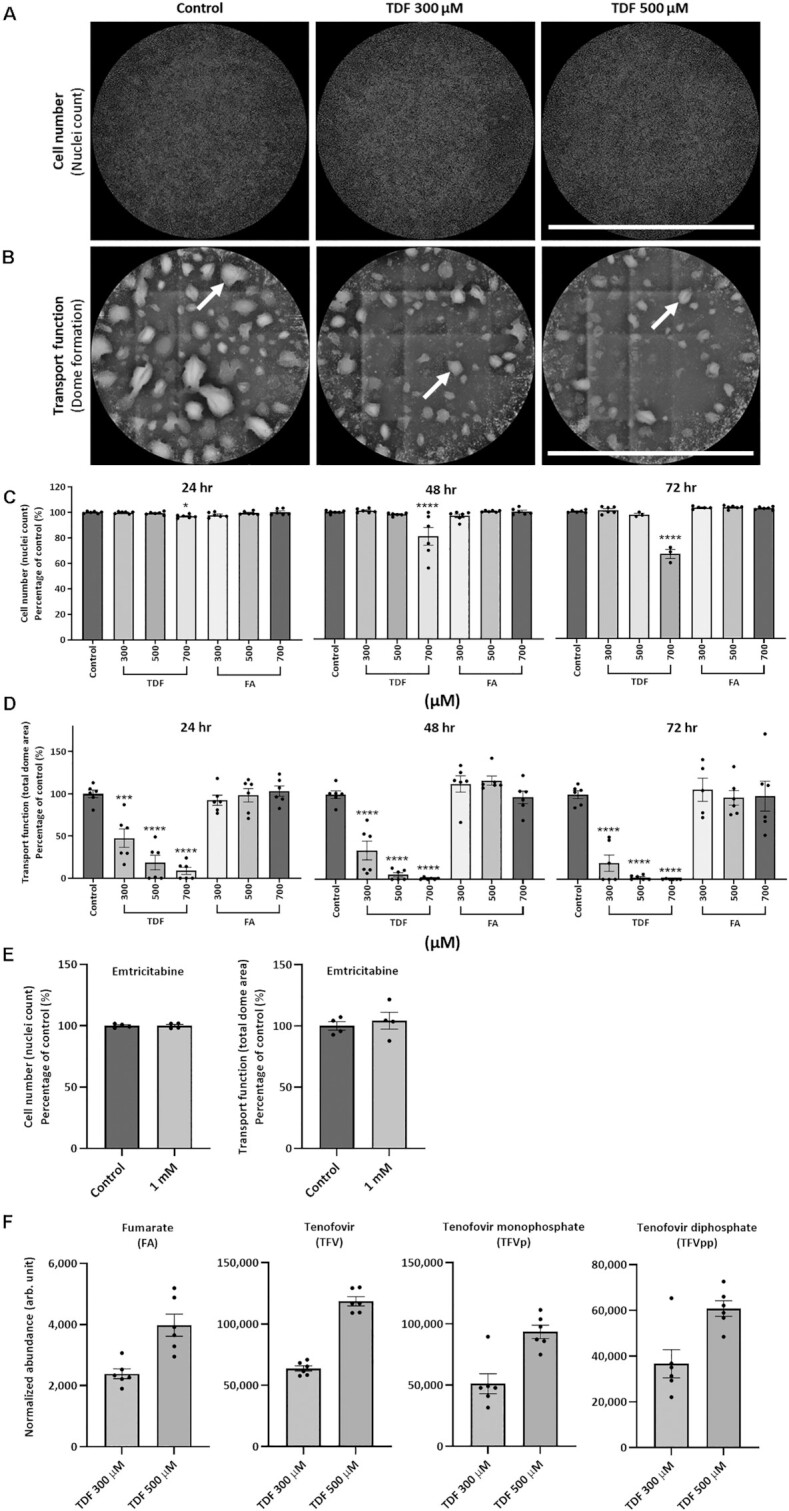
Tenofovir disoproxil fumarate (TDF) provokes dose- and time-dependent defects in solute transport function. Representative **(A)** composite images and **(B)** topography height-maps of cell monolayers exposed to 300, 500, or 700 μm TDF for 24 h. Cell monolayers were analyzed in the same manner following treatment with fumaric acid (FA). Scale bar = 1650 µm. Quantification of **(C)** cell number and **(D)** transport function (white arrows; domes) following drug exposure. (*n* = 6, mean ± SEM, *P < .05, ***P < .001, ^****^P < .0001, one-way ANOVA with Dunnett’s multiple comparisons test). **(E)** Quantification of cell number and transport function following 24-h treatment with Emtricitabine (*n* = 4, mean ± SEM, unpaired two-tailed *t*-test). **(F)** Targeted metabolomics analysis confirmed that TDF underwent appropriate enzymatic activation in treated cells, as indicated by the presence of the fumarate salt (FA), the active ingredient tenofovir (TFV), the monophosphate intermediate TFV monophosphate (TFVp), and the active antiviral metabolite TFV diphosphate (TFVpp) (*n* = 6, mean ± SEM).

Having established the pipeline, we exposed cells to different treatment concentrations of the prodrug TDF, since the active ingredient TFV has a very low cell permeability^36^ (TFV enters PT cells in vivo via basolateral OAT1/3 transporters,^24^ but we were not able to model this in our system). We found that TDF induced a dose- and time-dependent decrease in total dome area, without decreasing cell number, consistent with a functional effect on solute transport (Figure 2A–D). We also treated cells with fumarate alone, since fumaric acid esters have been associated with nephrotoxicity,^37^ but did not observe any deleterious effect on solute transport (Figure 2C–D), suggesting it is not responsible for TDF toxicity. Moreover, treatment for 24 h with 1 m m Emtricitabine, a cytidine analogue nucleoside reverse-transcriptase inhibitor, did not affect total dome area, indicating that the TDF effect is specific (Figure 2E). Importantly, we were able to identify the active metabolite TFVpp within TDF-treated cells, along with fumarate (Figure 2F), confirming that the prodrug was taken up and metabolized.

**Figure 3.**
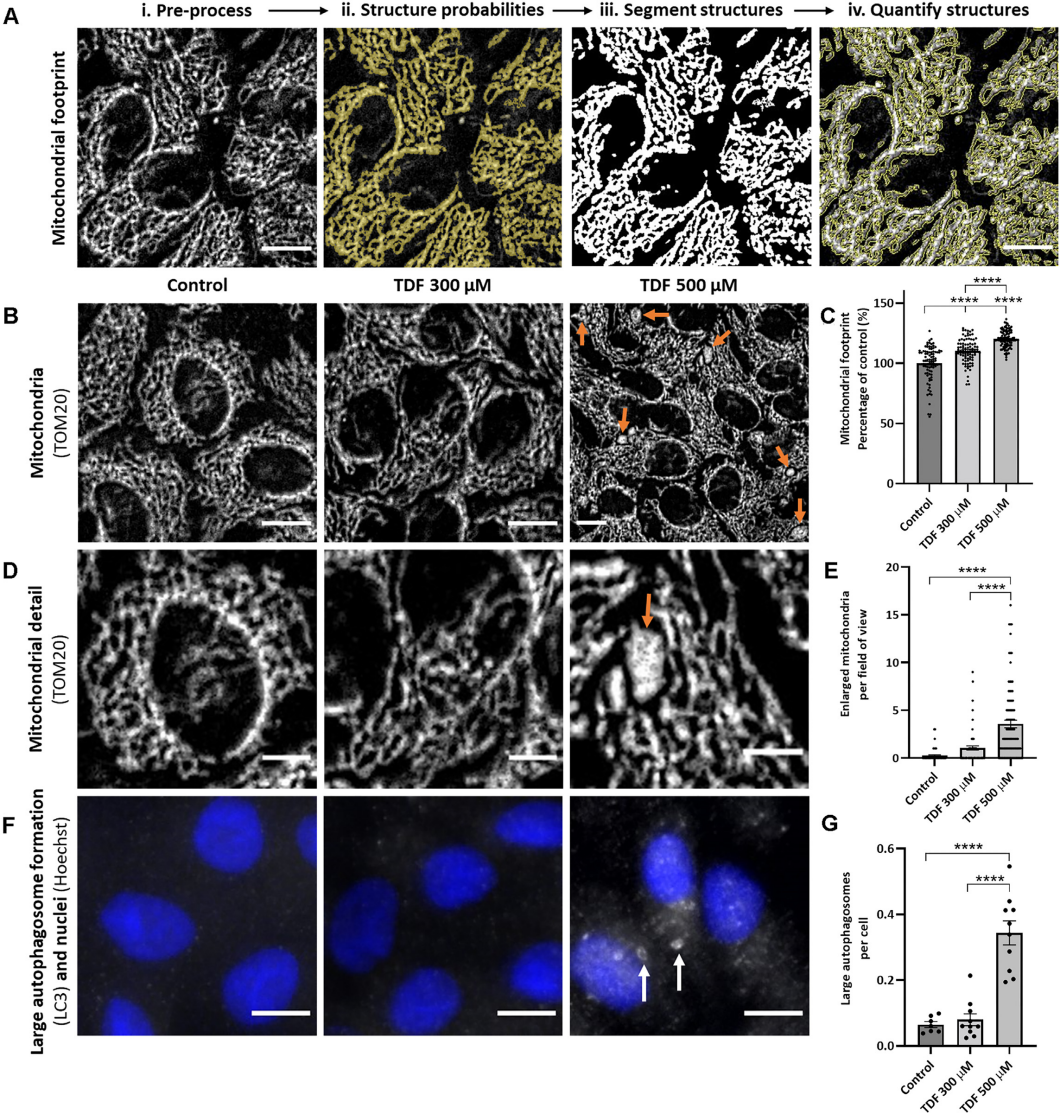
Generating representative in-vitro models of human TDF toxicity. **(A)** Image z-stacks were generated using our high-throughput imaging pipeline. Machine learning software was used to automatically identify, segment, and quantify mitochondria. Furthermore, with training and size thresholding, machine learning software was able to distinguish between mitochondria that were regular or enlarged in size. Scale bars = 10 μm. Representative immunofluorescence images and quantifications of mitochondria and autophagosomes following 24-h exposure to 300 or 500 µm TDF: **(B)** mitochondria and **(C)** mitochondrial footprint (total area of an image occupied by mitochondria); **(D)** mitochondrial morphology—including enlarged, dysmorphic mitochondria (orange arrows) interspersed with mitochondria displaying a normal morphology—and **(E)** quantification of enlarged mitochondria (*n* = 90 fields of view—48 × 48 μm—from 3 samples, mean ± SEM, ^****^P < .0001, one-way ANOVA with Tukey’s multiple comparisons test); **(F)** large autophagosome formation (white arrows) and **(G)** quantification (*n* = 10 fields of view—331.5 × 331.5 μm—from three samples, mean ± SEM, ^****^P < .0001, one-way ANOVA with Tukey’s multiple comparisons test). Scale bars for tiles labeled “mitochondria” and “large autophagosome formation” = 10 μm. Scale bars for tiles labeled “mitochondrial detail” = 5 μm.

Next, we assessed mitochondrial morphology by immunofluorescence staining for the outer membrane marker TOM20. Images were analyzed using a modified version of an established machine learning pipeline (Figure 3A).^38,39^ This revealed that TDF induced dose-dependent changes in the total mitochondrial footprint within cells (Figure 3B–>C), and increased the prevalence of individually enlarged, dysmorphic mitochondria (Figure 3D–E). Cells were also stained with LC3 to assess activation of autophagy, as another marker of metabolic stress (Figure 3F–G).

From the above experiments, we identified two treatment regimens that recreated the major clinical phenotypes described in patients. First, cells exposed to 300 µm TDF for 24 h displayed a partial inhibition of transport function (Figure 2), but without evidence of major changes in mitochondrial morphology or activation of autophagy (Figure 3), and thus resembled patients with isolated transport defects. Second, cells treated with 500 µm TDF for 24 h developed a more drastic loss of transport (Figure 2), which was associated with the appearance of enlarged, dysmorphic mitochondria and activation of autophagy (Figure 3), and therefore represented a model of severe toxicity.

These two models were then used for further experiments to elucidate underlying changes in metabolism responsible for the phenotypes. By focusing on an early (24 h) time point, we aimed to detect direct toxic effects of TDF, rather than compensatory responses. The introduction of a purine nucleotide analog could potentially induce multiple perturbations within living cells, which might not be adequately captured by any one single assay. We therefore instigated a comprehensive approach using several different orthogonal readouts and integrated data from each.

### Metabolomic Screening of TFV-treated Cells

**Figure 4.**
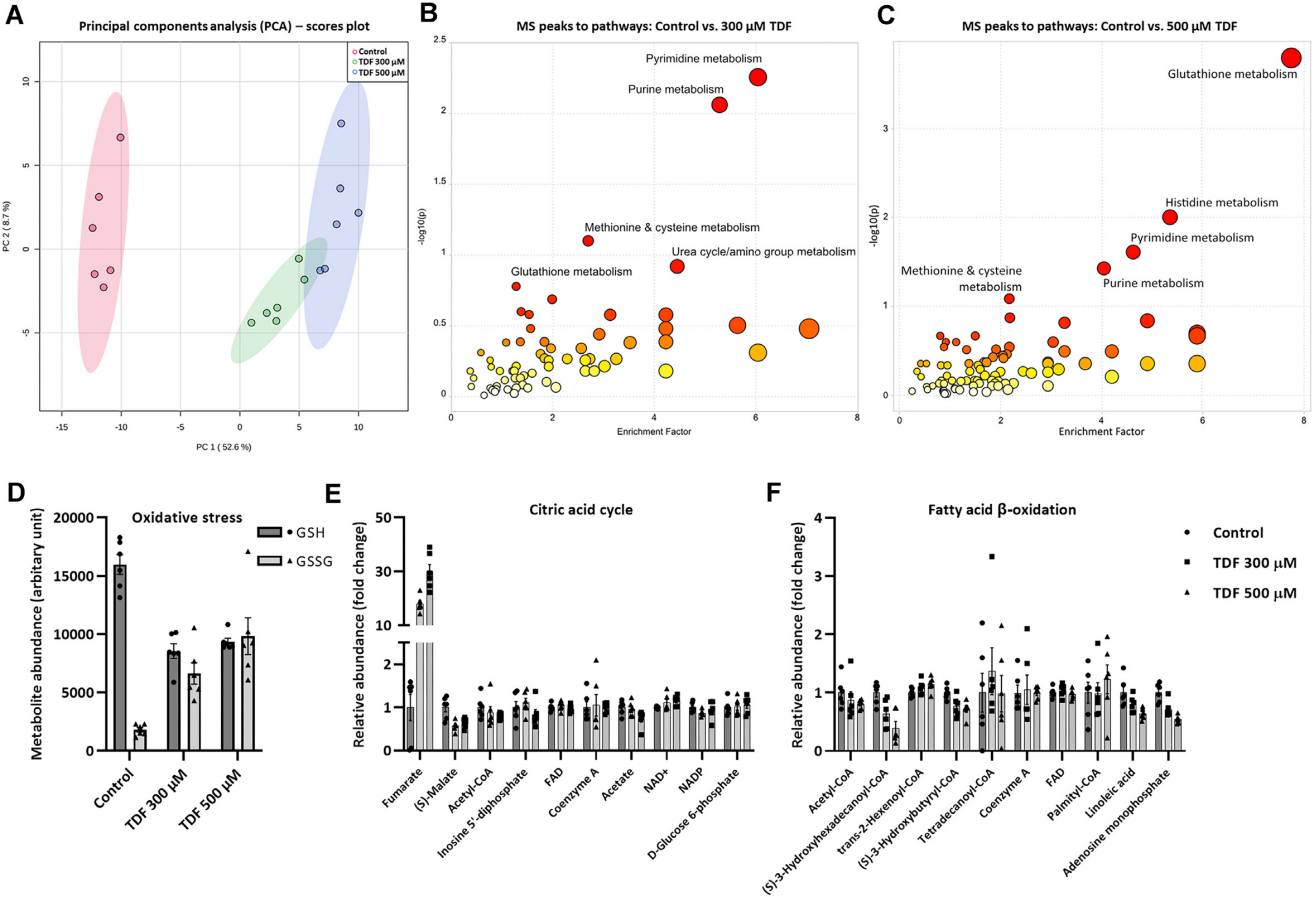
The effects of TDF on the cellular metabolome.**(A)** Principal components analysis (PCA) of untargeted metabolomics data (colored shaded areas indicate the 95% confidence regions) (*n* = 6). Unbiased MS peaks to pathways analysis (of the top 10% most changed peaks) following 24-h exposure to **(B)** 300 or **(C)** 500 µm TDF revealed major changes in glutathione, pyrimidine, and purine metabolism. The five most disrupted pathways are indicated in the plots. **(D)** Oxidative stress was evaluated by assessing the level of reduced glutathione (GSH) relative to that of oxidized glutathione (GSSG) (*n* = 6, mean ± SEM). Relative abundance of metabolites with an assigned identity involved in the **(E)** citric acid cycle and **(F)** fatty acid *β*-oxidation (*n* = 6, mean ± SEM).

First, to gain an overview of the effects of TDF on cellular metabolism, we performed metabolomic screening using MS. Principal component analysis (PCA) of the entire dataset revealed clear separation of treated cells from controls, with a more modest distinction between the two treatment groups (Figure 4A). Unbiased pathway activity analysis revealed major changes in purine/pyrimidine metabolism, further confirming uptake of a purine nucleotide analog (Figure 4B–C). We also found evidence of severe oxidative stress (Figure 4B–D), as reported previously in animal models.^20^ In contrast, we did not observe any substantial changes in several major mitochondrial pathways, including the citric acid cycle (aside from an increase in fumarate) (Figure 4E) and beta oxidation of fatty acids (Figure 4F), suggesting that the effects of TDF on mitochondria are quite targeted and specific.

### The Active Metabolite of TFV Inhibits Complex V Activity

**Figure 5.**
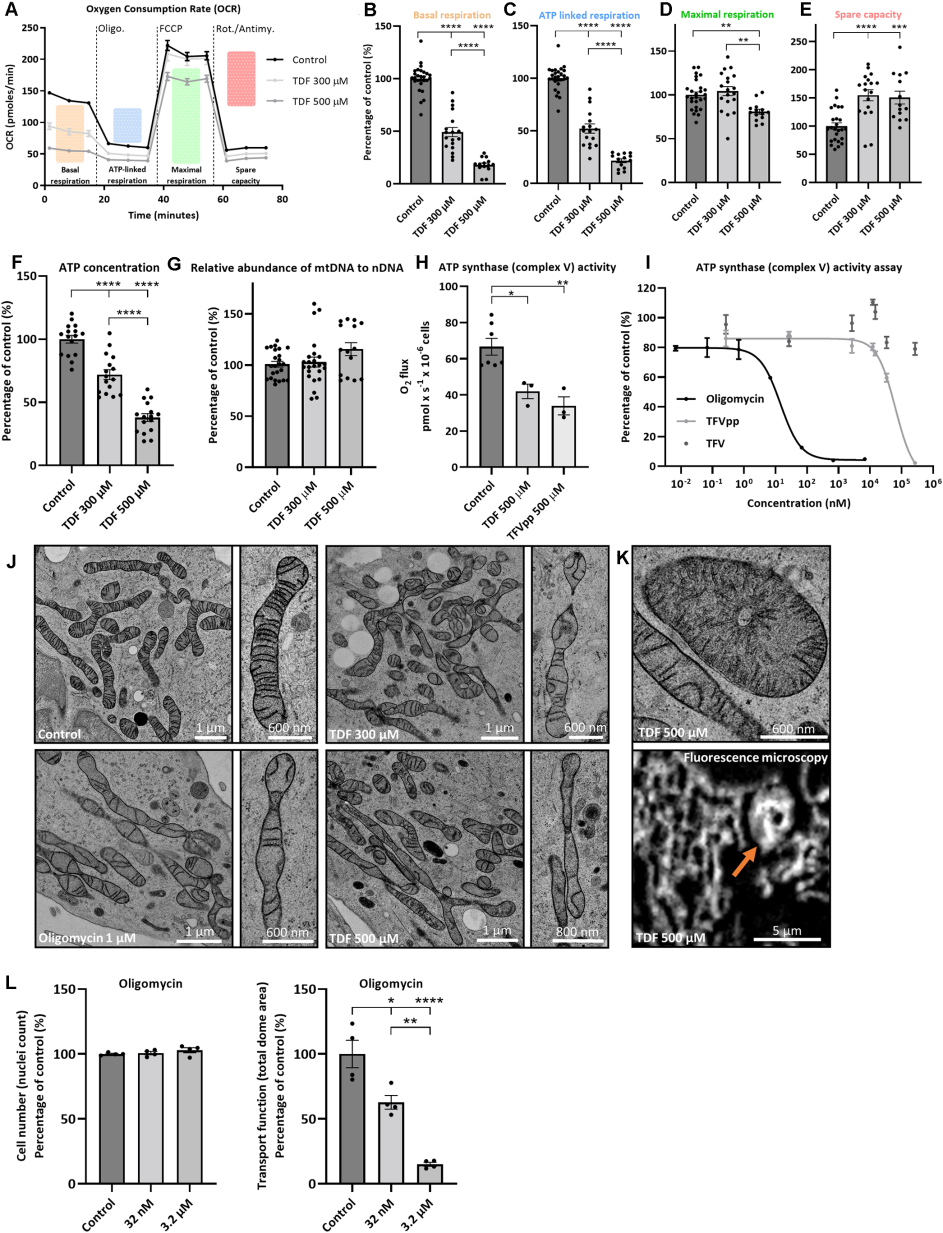
Tenofovir disoproxil fumarate induces a loss of complex V (ATP synthase) activity.**(A)** Raw OCR traces following 24-h treatment with TDF (*n* of treatment groups = 15, *n* of control group = 30, mean ± SEM). Modulators of the respiratory chain reveal key parameters of mitochondrial function: **(B)** basal respiration; **(C)** ATP-linked respiration; **(D)** maximal respiration; and **(E)** spare respiratory capacity (*n* of treatment groups = 15, *n* of control group = 30, mean ± SEM, **P < .01, ***P < .001, ^****^P < .0001, one-way ANOVA with Tukey’s multiple comparisons test). **(F)** Cellular ATP levels following 24-h TDF treatment (*n* = 16, mean ± SEM, ^****^P < .0001, one-way ANOVA with Tukey’s multiple comparisons test). **(G)** Relative abundance of mitochondrial DNA (mtDNA) to nuclear DNA (nDNA) after 24-h TDF exposure (*n* = 24 [eight samples analyzed using three different primers for mtDNA genes], mean ± SEM, one-way ANOVA with Tukey’s multiple comparisons test). **(H)** Complex V activity in cells exposed to 500 µm TDF for 24 h or 500 µm TFVpp for 30 min (*n* = 3, mean ± SEM, *P < .05, **P < .01, one-way ANOVA with Tukey’s multiple comparisons test). **(I)** Complex V activity following 30-min exposure to TFVpp, TFV, or Oligomycin (*n* = 3, mean ± SEM). **(J)** Representative electron micrographs. Mitochondria of cells treated with Oligomycin (30 min) or TDF (24 h) displayed a loss of normal cristae formation, in the absence of changes in matrix density (*n* = 3). **(K)** Mitochondria of cells treated with TDF revealed distinct enlarged, dysmorphic, doughnut-shaped mitochondria with widened cristae folds. Similar shaped mitochondria were also observed in cells stained for TOM20 and imaged using fluorescence microscopy (orange arrow). **(L)** Quantification of cell number (nuclei count) and transport function (total dome area) following treatment with Oligomycin for 24 h (*n* = 4, mean ± SEM, *P < .05, **P < .01, ^****^P < .0001, one-way ANOVA with Dunnett’s multiple comparisons test).

Next, to assess RC function, we performed measurements of oxygen consumption rate (OCR) in cells treated with TDF. This revealed a dose-dependent decrease in both baseline OCR and ATP-linked OCR (Figure 5A–C). However, spare respiratory capacity and maximal OCR were relatively well preserved, suggesting that short-term exposure to TDF does not exert any major rate limiting influence on either the supply of substrates to the RC (consistent with the metabolomics data), or the activity of complexes I–IV (Figure 5A, D, and E). Tenofovir disoproxil fumarate also caused a dose-dependent decrease in cellular ATP levels after 24 h (Figure 5F). In contrast, we did not observe any reduction in mtDNA abundance (Figure 5G).

These findings prompted us to consider an effect on the activity of complex V, which is responsible for generating ATP within mitochondria. Of note, the active metabolites TFVp and TFVpp are structural analogs of ADP and ATP, respectively, which are the substrates for complex V. We observed direct evidence of decreased complex V activity in cells incubated with TDF for 24 h, and also in permeabilized cells acutely treated with TFVpp (Figure 5H). Moreover, TFVpp produced a dose-dependent inhibition of isolated complex V activity (Figure 5I).

Since dimers of complex V play a crucial role in maintaining normal cristae folds,^40^ we performed high-resolution electron microscopy to examine mitochondrial ultrastructure. This revealed severe cristae abnormalities in cells treated with TDF that were indistinguishable from those induced by the known complex V inhibitor oligomycin (Figure 5J and K and Figure S1). Moreover, cristae changes also closely resembled ultrastructural features previously reported in humans with TDF toxicity.^16^ Finally, treating cells with oligomycin also decreased dome area in a dose-dependent manner (Figure 5L), thus replicating the functional effects of TDF.

In summary, our findings suggest that the major acute effect of TDF on mitochondria is loss of complex V function, likely due to inhibition from the active metabolite (TFVpp), leading to severe alterations in cristae structure and a decrease in cellular ATP, and a secondary defect in active solute transport.

### Gene Expression Analysis in TFV-treated Cells

**Figure 6.**
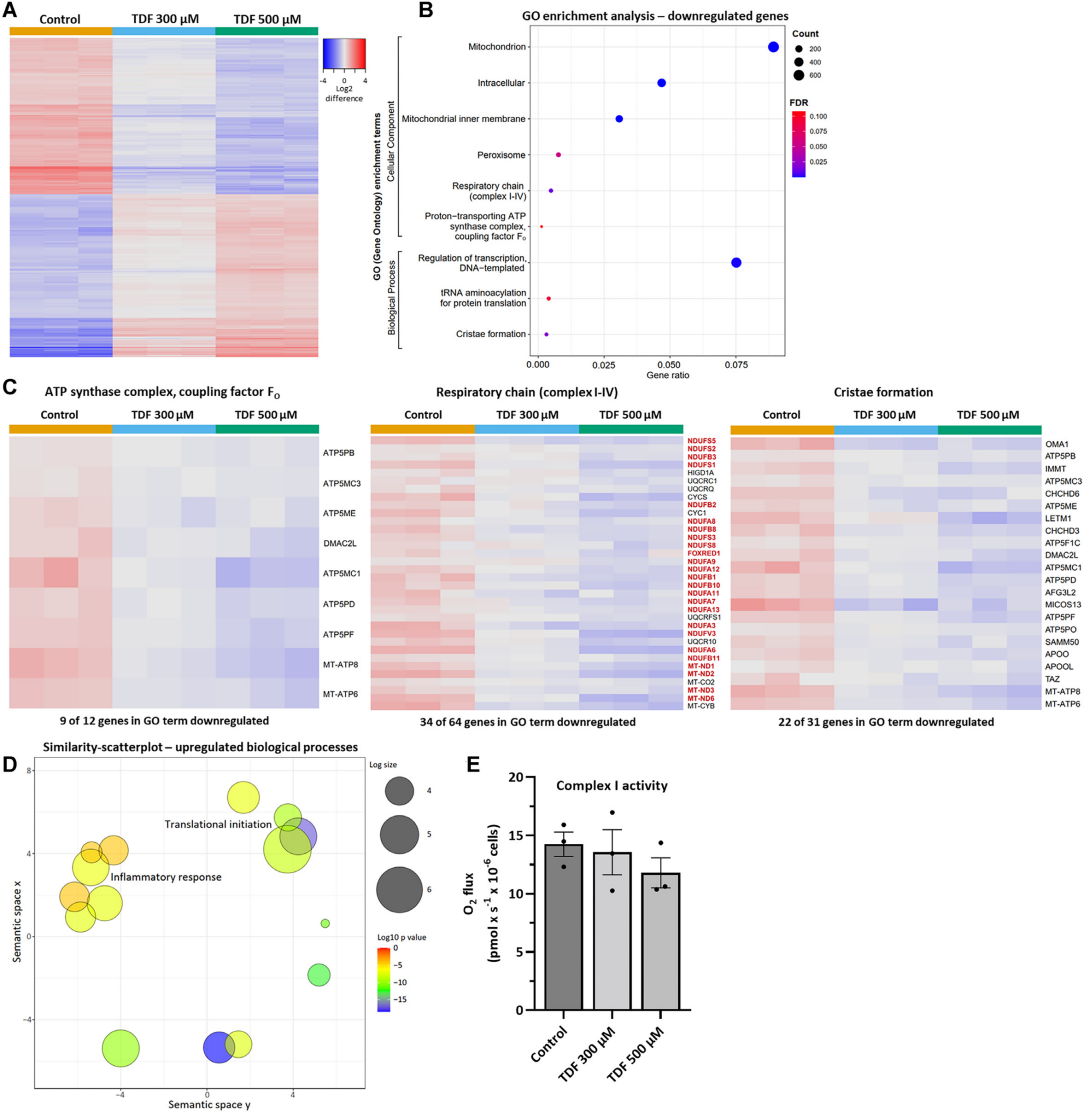
Tenofovir disoproxil fumarate induces downregulation of mitochondrial genes.**(A)** Heatmap illustrating the dose-dependent expression of the 2 000 most changed genes following 24-h treatment with 300 or 500 µm TDF (*n* = 3). Red denotes higher expression and blue lower. **(B)** Unbiased gene ontology (GO) enrichment analysis of RNA-sequencing data revealed a specific downregulation of mitochondrial related genes. “Count” indicates the number of genes that are differentially expressed for a given GO term. The “FDR” (false detection rate) is a *P*-value that has been adjusted for multiple comparisons. “Gene ratio” is the number of differentially expressed genes for a given GO term in relation to the total number of differentially expressed genes. **(C)** Tenofovir disoproxil fumarate provoked a downregulation of genes involved in the F_0_ subunit of complex V, complex I of the respiratory chain (marked in red), and mitochondrial cristae formation. **(D)** Similarity-scatterplot—of GO enrichment analysis—revealed an upregulation of genes associated with the overarching umbrella terms “inflammatory response” and “translational initiation.” **(E)** Complex I activity measured in the presence of the NADH-linked substrates glutamate (10 m m) and malate (2 m m) following 24-h treatment with 300 or 500 µm TDF (*n* = 3, mean ± SEM, one-way ANOVA with Dunnett’s multiple comparisons test).

Having assessed the functional effects of TDF on mitochondria, we next proceeded to investigate alterations in gene expression with RNA-Seq. This revealed significant differences in the transcriptome of cells treated with TDF for 24 h. When compared to controls, cells exposed to 300 µm TDF had 5 055 differentially expressed genes (*P*-value threshold ≤ .01, log ratio threshold ≥ 0.5), whilst cells treated with 500 µm TDF had 7 026 (Figure 6A).

Gene ontology (GO) enrichment analysis indicated that the vast majority of downregulated genes localized to mitochondria, and in particular the inner membrane, providing further evidence that TDF specifically disrupts these organelles. Moreover, complex V and cristae formation were identified as substantially altered cellular components and processes, respectively, with the relevant genes displaying dose-dependent suppression (Figure 6B–C and Figures S2–S4). Thus, the functional and morphological effects of TDF on mitochondria are closely reflected at the level of gene expression.

The expression of complex I subunits was also decreased by TDF exposure (Figure 6C). In contrast, genes involved in inflammatory pathways and translational initiation—probably reflecting a cell stress response^41^—were markedly upregulated (Figure 6D). In view of the effect on complex I gene expression, we performed OCR experiments in permeabilized cells provided with glutamate/malate (to assess complex I activity), but did not observe any differences between control and TDF-treated cells (Figure 6E). Thus, our results suggest that the predominant acute effect of TDF is on the function of complex V.

### Integrated Network Analysis of Metabolomics and Transcriptomics (Multi-Omics)

Since metabolomic and transcriptomic screens can highlight perturbations in pathways that might functionally interact, integrating results from both (multi-omics) can provide additional insights into the critical shared factors that drive the overall phenotype.^42^ We therefore performed an integrated network analysis of our data, to visualize interactions between genes and metabolites.^43^ This confirmed that TDF induces major changes in purine/pyrimidine metabolism (Figure S5A–B). Crucially, extensive connectivity was established between ADP/ATP and changes in enzyme expression and metabolite abundance (Figure S5A–D), showing that these molecules act as important nodes within the interactome. Given that complex V plays a decisive role in determining ADP/ATP levels, these results further suggest that suppression of complex V activity is a pivotal event in TDF toxicity.

### Generation of a Chronic TFV Treatment Cell Model

**Figure 7.**
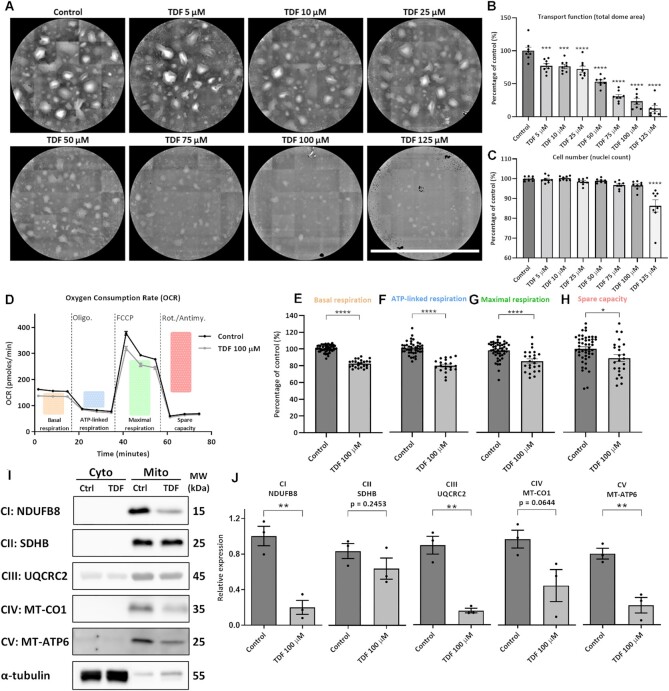
Generating and characterizing a model of chronic TDF toxicity.**(A)** Representative height-maps of cell monolayers exposed to 5–125 μm of TDF for 9 d. Scale bar = 1650 µm. **(B)** Dome formation (total dome area) was used to assess transport function, whilst **(C)** nuclei were counted and applied as a measure of cell number (*n* = 8, mean ± SEM, ***P < .001, ^****^P < .0001, one-way ANOVA with Dunnett’s multiple comparisons test). **(D)** Raw OCR traces (*n* of treatment group = 24, *n* of control group = 48, mean ± SEM) and **(E–H)** key parameters of mitochondrial respiration following 9-d treatment with 100 µm TDF (*n* of treatment group = 24, *n* of control group = 48, mean ± SEM, *P < .05, ^****^P < .0001, unpaired two-tailed *t*-test). **(I)** Representative western blots and **(J)** quantifications of respiratory chain subunit protein abundance from isolated mitochondria of cells treated with 100 µm TDF for 9 d (*n* = 3, mean ± SEM, nonsignificant = *P* > .05, **P < .01, unpaired two-tailed *t*-test).

Having identified the acute impact of TDF on mitochondria, we next explored more chronic effects by treating RPTEC/TERT1 cells for an extended time (9 d) and at lower doses (5–125 µm). We again observed a dose-dependent effect of TDF on solute transport (Figure 7A–C), and decreases in both baseline and ATP-linked OCR (Figure 7D–F). Although maximal OCR was slightly lower than controls, substantial reserve capacity was still present (Figure 7D, G–H). Moreover, western blotting confirmed a severe decrease in complex V subunit expression (and complex I), but no change in complex II, and smaller decreases in complexes III and IV (Figure 7I and J). Thus, loss of complex V activity/expression remains a major phenotype in a chronic model of TDF toxicity.

### Validation of Results in Humans With TFV Toxicity

**Figure 8.**
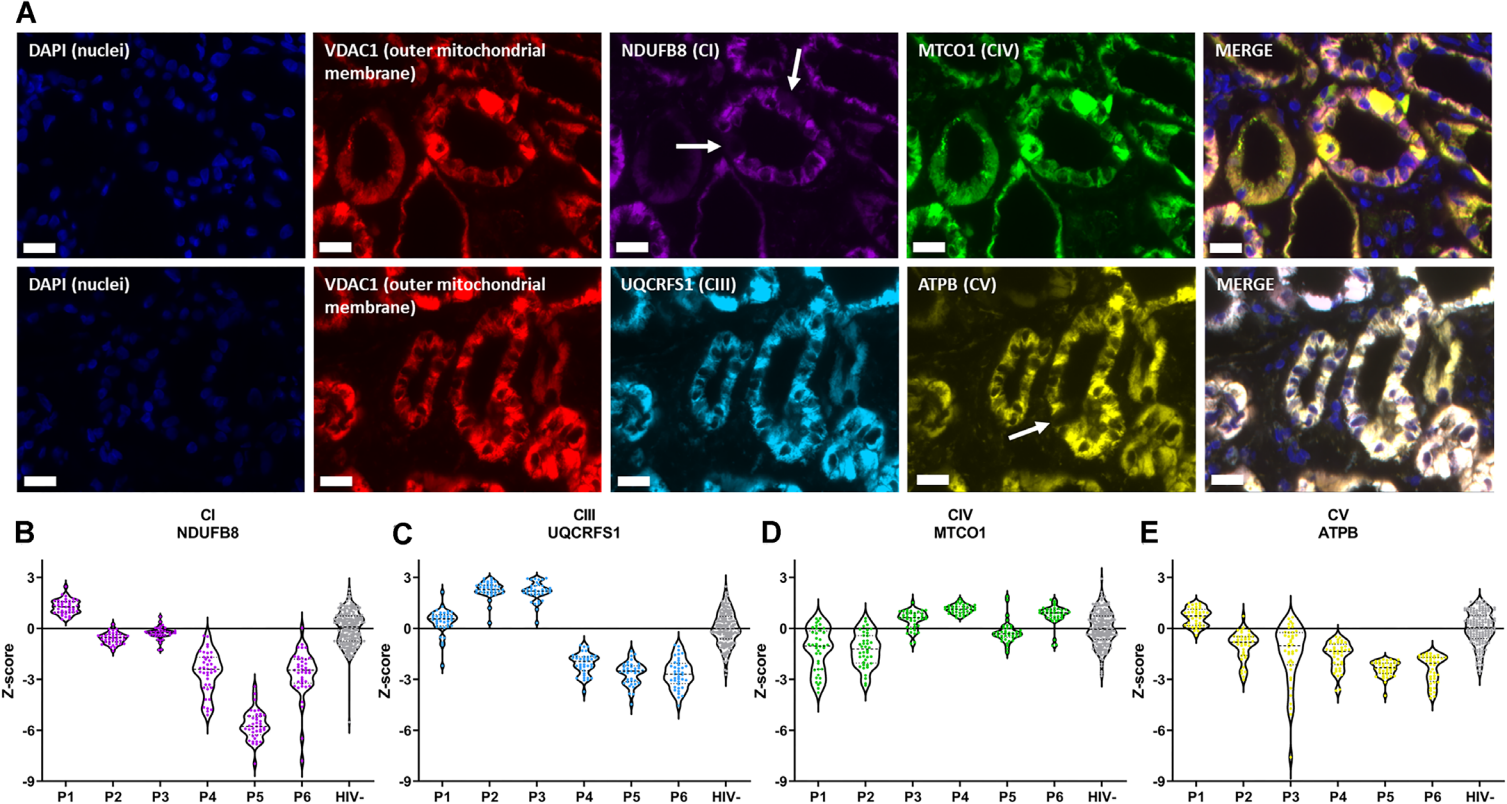
Immunofluorescence staining of respiratory chain complex expression in kidney biopsy samples from patients with TDF toxicity.**(A)** Example images from a TDF-treated case, with markers for DAPI (nuclear marker), VDAC1 (mitochondrial mass), NDUFB8 (complex I subunit), MTCO1 (complex IV subunit), UQCRFS1 (complex III subunit), and ATPB (complex V subunit). White arrows indicate individual PT epithelial cells with deficiency in the respective complex. Scale bar = 20 µm. **(B–E)** Violin plots depicting the Z-scores for protein levels of each complex in individual PTs from TDF-treated cases (Patient 1 [P1]–Patient 6 [P6]), relative to the levels from five pooled HIV-negative (HIV-) controls: **(B)** complex I, **(C)** complex III, **(D)** complex IV, and **(E)** complex V. Each dot represents an individual PT (*n* = 40).

Finally, we performed antibody staining in kidney biopsies from humans with TFV toxicity to validate our in-vitro findings. (Figure 8). At the point of biopsy, all people with HIV were on an antiretroviral therapy regimen that currently or recently (within 1 mo) included TDF (Table S1). All cases had histological evidence of acute tubular injury (ATI) and were compared with HIV negative controls with normal tissue. Multiplex immunofluorescence staining demonstrated deficiency of RC complexes (I, III, IV, V) within PT segments from cases with ATI. Notably, deficiency of complex V was most commonly observed, followed by complex I (Figure 8), similar to findings in our cell model. In contrast, expression of complex IV was better preserved.

## Discussion

Nephrotoxicity is a substantial cause of kidney disease, but identifying cellular mechanisms has proven a considerable challenge. This is exemplified by TDF, which causes tubular damage for reasons that have been unexplained for more than 20 yr. To disentangle the pathogenesis of TDF toxicity, we overcame two major practical hurdles. First, we developed a new high-throughput imaging screen to rapidly assess solute transport and mitochondrial morphology in human-derived cells, and leveraged this to generate realistic in-vitro disease models. Second, we used multiparametric metabolic profiling to define the pivotal molecular changes that underpin the phenotype. From this, we identified that TDF inhibits and suppresses complex V in a dose-dependent manner, which provides a new paradigm for understanding well-described features of toxicity in humans (Figure S6—summary figure).

Various approaches have been used to evaluate solute transport in epithelia, including tagging with fluorescent labels, but applying these in a high-throughput manner is challenging. We demonstrate that automated imaging of dome formation represents a convenient and robust method, which could easily be integrated into preclinical screens. Moreover, although oxygen consumption measurements, metabolomics, and transcriptomics are all powerful tools, they each provide a different snapshot of cellular metabolism. By integrating results from all three, we show it is possible to build a more holistic picture of metabolic disturbances and pinpoint their proximate causes.

The critical new insight gained from this approach was that the active metabolite TFVpp inhibits complex V, and that TDF causes a rapid and dose-dependent decrease in complex V function and expression, at a time point when RC function is otherwise relatively well maintained. Moreover, detailed metabolomic analysis excluded adverse effects on other major pathways, such as the citric acid cycle, further underlining the specificity of this phenomenon, and excluding a generalized mitochondrial toxicity. Downregulation of complex V expression was confirmed at protein level in a chronic model and in human biopsies. Some older antiviral drugs cause mtDNA depletion,^21^ but in such a scenario, we would expect a more prominent effect on complex IV, which was comparatively preserved. Furthermore, at the time of complex V inhibition, we did not detect a decrease in mtDNA abundance.

Because PT cells in vivo are almost entirely dependent on aerobic metabolism to generate ATP to power solute movement, loss of complex V activity readily explains why patients with TDF toxicity present with transport defects. Our data suggest that TFVpp is a relatively weak inhibitor of complex V, but this is not unexpected, since it is extremely unlikely that a more potent inhibitor could be tolerated. Moreover, purine nucleotides can inhibit complex V by a variety of different mechanisms,^44^ and it is conceivable that analogs like TFVpp might also indirectly lower complex V activity (eg, by limiting uptake of purine nucleotides into mitochondria), which could amplify the toxic effect.

Dimers of complex V maintain the folded architecture of cristae, which in turn is crucial for ensuring optimal positioning of RC complexes.^40^ Thus, by identifying that TDF targets complex V, we provide an explanation for the prominence of cristae abnormalities in TDF toxicity.^16–18^ Interestingly, studies in humans with hereditary defects in complex V genes—including MT-ATP6 (heavily downregulated in our study)^45^—have described very similar morphological changes in mitochondria to those induced by TDF.^46^

Inhibition of complex V activity is particularly hazardous for cells, since it simultaneously decreases ATP supply and increases the risk of reactive oxygen species (ROS) production by hyperpolarizing the inner membrane. We note a previous study reporting that TFV increases mitochondrial superoxide production.^36^ We also observed a decrease in complex I expression, which has been reported in rodents,^47^ but we cannot discern from our experiments whether this is a direct or indirect effect of TDF. Complex I is a potent source of ROS generation, and an intriguing recent study suggested that decreasing its activity is beneficial in the presence of a complex V inhibitor.^48^ Thus, downregulation of complex I might represent a protective response to loss of complex V. Tenofovir disoproxil fumarate also induced upregulation of genes involved in inflammatory pathways, which may contribute to the interstitial fibrosis that occurs in patients with chronic toxicity.^16^

Our study has some potential limitations. Measurement of dome formation necessitated that cells were grown on an impermeable surface, rather than permeable cell culture inserts. This meant that drugs were applied from the apical side, whereas uptake of TFV in vivo is thought to be basolateral (mainly via OAT1/3).^24^ Moreover, we used the prodrug TDF to ensure uptake and conversion to the active metabolite TFVpp within cells. We also cannot determine from our imaging screen how exactly TDF decreases solute transport and dome formation, but an effect on ATP supply seems most likely, given a similar effect of oligomycin. In addition, although high-throughput screening in principle provides the opportunity to test therapeutic interventions, we found that TDF toxicity was very difficult to prevent or reverse (not shown), underlining the centrality of complex V to normal cell function. Finally, a newer formation of tenofovir (tenofovir alafenamide) might be less nephrotoxic than TDF in the short term, although longer term benefits are unclear, and the active ingredient is the same.^49^

Previous studies have suggested that TDF toxicity is dose dependent.^50^ In the present study, to study the acute effects of TDF toxicity, we used concentrations that are higher than typical blood levels of TFV in healthy people.^51^ However, TDF toxicity in humans is a cumulative phenomenon,^26^ likely explained by accumulation of harmful metabolites within PT cells over time. Accordingly, risk factors for toxicity—including renal impairment, polymorphisms in drug transporters,^52^ and concomitant usage of medications that compete for efflux pathways^53^—all promote tubular drug/metabolite aggregation. Therefore, high doses of TFV/TDF are needed to induce acute toxicity in cell^36^ or animal models,^54^ and the concentrations we used are comparable to blood TFV levels (C_max_ 219.3 µm, AUC 488.3 µm∙h/mL) reported in nonhuman primate studies.^54^ Moreover, differences in intracellular conversion rate of TFV to the active metabolite mean that appropriate doses for in-vitro studies are likely to be model specific.

Given the difficulties of extrapolating drug doses from in-vivo conditions, we focused instead on determining which experimental protocols reproduced in vitro the characteristic features of functional toxicity in patients. Importantly, in both acute (higher dose) and chronic (lower dose) models, we identified complex V dysfunction as the prominent feature, and by applying comprehensive metabolic screening, we were able to exclude major perturbations in other pathways. Nevertheless, we acknowledge that our findings should ideally be replicated in the future, when model systems are available that fully reflect the complexity of TDF metabolism and transport in living organisms.

In summary, by combining high-throughput imaging with detailed metabolic profiling, we were able to identify for the first time the mitochondrial phenotype of TDF toxicity in renal epithelial cells. We envisage that this approach could be harnessed not only to investigate other drugs and toxins, but also for further purposes, such as uncovering novel genetic regulators of transport and metabolism in epithelia.

## Supplementary Material

zqac065_Supplemental_File

## Data Availability

The data underlying this article will be shared on reasonable request to the corresponding author.
