# Supplementary material for: Integration of High-Throughput Imaging and Multiparametric Metabolic Profiling Reveals a Mitochondrial Mechanism of Tenofovir Toxicity
Source: Function (Oxf). 2022 Dec 24;4(1):zqac065. doi: 10.1093/function/zqac065 (PMC9840465; doi:10.1093/function/zqac065)
Supplement: zqac065_Supplemental_File [file zqac065_supplemental_file.docx]

## Integration of high-throughput imaging and multi-parametric metabolic profiling reveals a mitochondrial mechanism of tenofovir toxicity

## Supplementary Materials

## Supplementary Data

Supplementary Figure 1. **Inhibition of complex V (mitochondrial ATP synthase) disrupts cristae architecture.** **(A)** Cristae in control mitochondria were positioned in tight folds (yellow arrows). **(B)** Cells exposed to the complex V inhibitor Oligomycin for 30 minutes developed widened cristae folds (white arrowheads) and mitochondrial morphology abnormalities. Cristae folds of cells treated for 24 hours with **(C)** 300 or **(D)** 500 µM TDF were indistinguishable from those incubated with Oligomycin. (n = 3).

Supplementary Figure 2. **Gene Ontology (GO) enrichment analysis. (A)** Downregulated and **(B)** upregulated GO enrichment terms following 24 hours treatment with 300 µM TDF.

Supplementary Figure 3. **Gene Ontology (GO) enrichment analysis. (A)** Downregulated and **(B)** upregulated GO enrichment terms following 24 hours treatment with 500 µM TDF.

Supplementary Figure 4. **Identities of downregulated genes associated with the Gene Ontology (GO) enrichment term ‘inner mitochondrial membrane’.** 24 hours treatment with 300 or 500 µM TDF downregulated the expression of genes involved in the inner mitochondrial membrane. TDF inhibited the expression of genes encoded by both the mitochondrial and nuclear genomes. (n = 3).

Supplementary Figure 5. **Gene-metabolite interaction network analysis demonstrates disruption of ATP and ADP following TDF exposure and illustrates the extensive interconnectedness of ATP and ADP with other differentially expressed genes and metabolites.** Disruption of ATP and ADP following 24-hour treatment with **(A)** 300 or **(B)** 500 µM TDF was linked to dysregulation of many other functionally related genes and metabolites. Furthermore, network analysis confirmed that TDF exposure induced major changes in purine and pyrimidine metabolism pathways, which were substantially more disrupted than any other pathway: metabolites (green squares) and genes (green circles) associated with purine metabolism; metabolites (yellow squares) and genes (yellow circles) associated with pyrimidine metabolism; blue squares = all other metabolites; pink circles = all other genes. Degree and betweenness – two established node centrality measures – were used to predict node (i.e. metabolite or gene) importance in cells treated with **(C)** 300 or **(D)** 500 µM TDF. The degree of a node indicates the number of connections it has to other nodes. Thus, nodes with a higher degree – such as ATP and ADP – are more important in a network. Betweenness centrality measures the number of paths going through the node and takes into consideration the global network structure. Nodes with higher betweenness act as important ‘bottlenecks’ in a network.


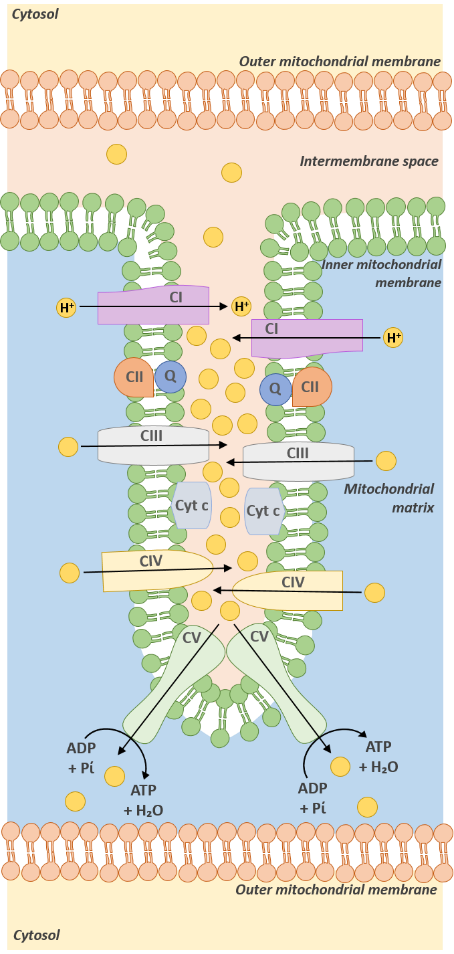

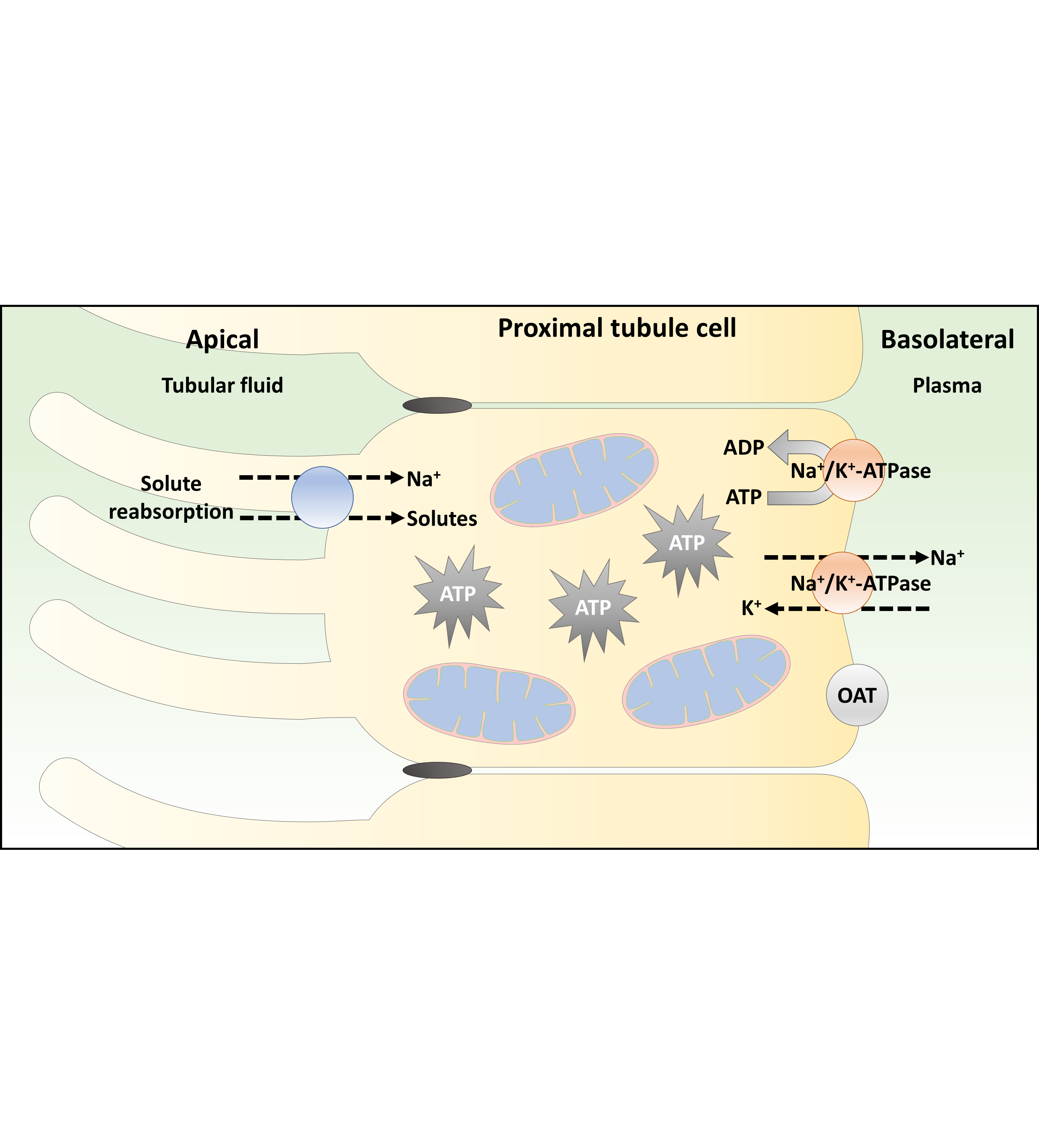

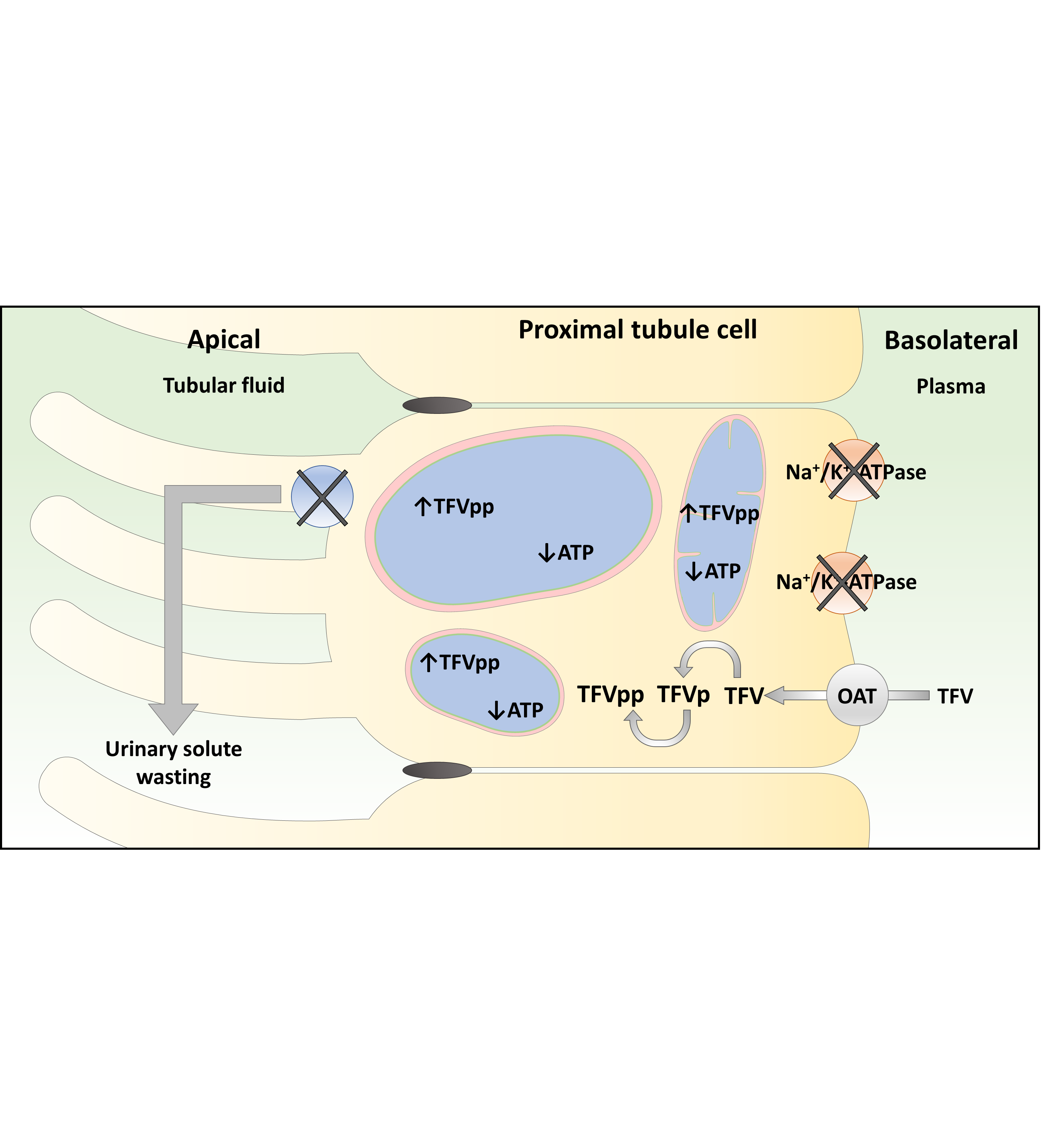

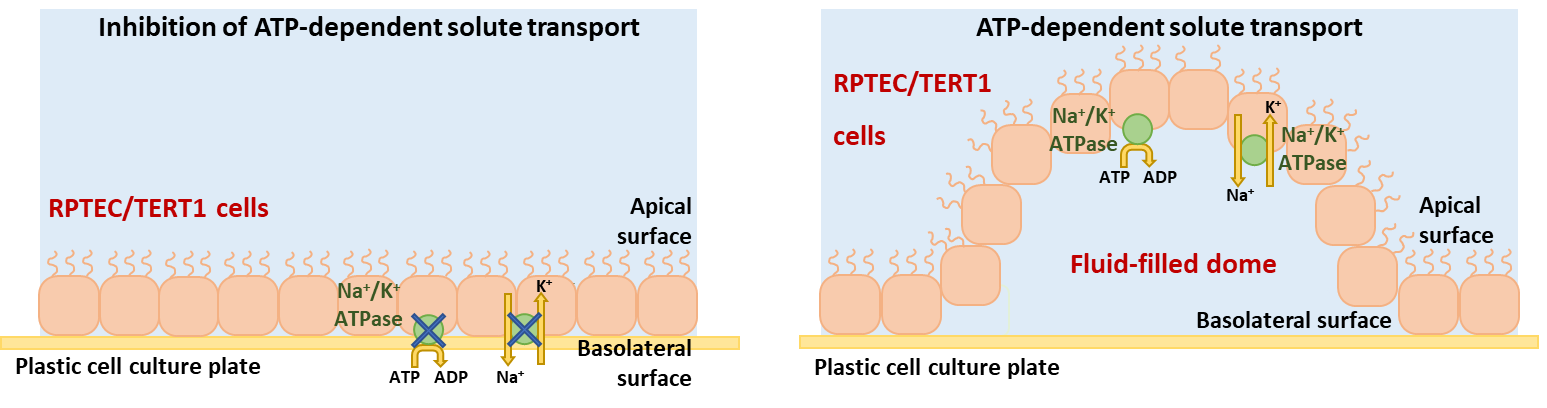

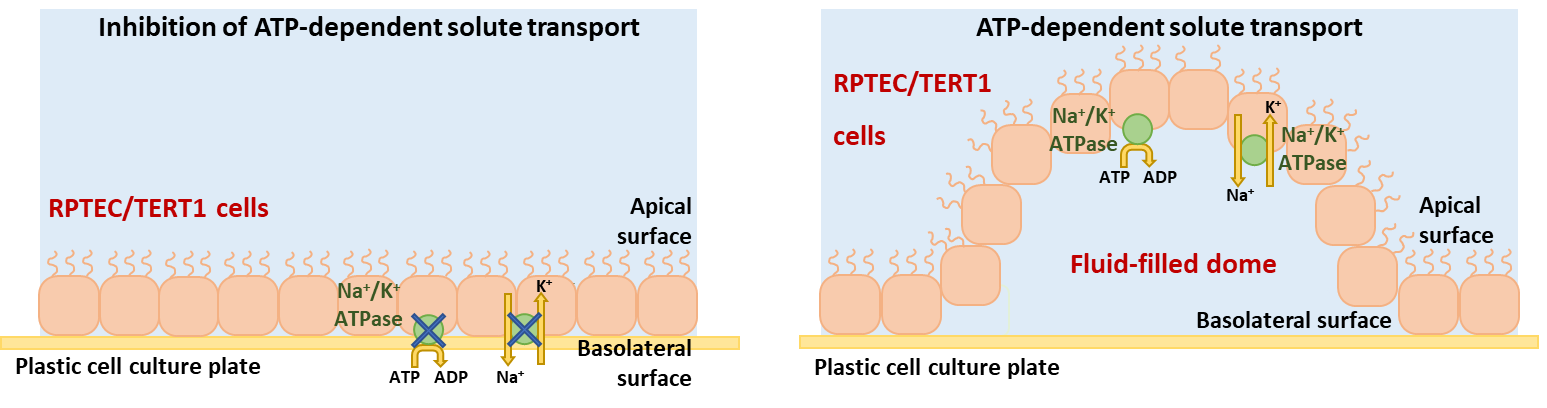


**Proximal tubule in Tenofovir disoproxil fumarate (TDF) toxicity**

**Proximal tubule under normal conditions**

**Mitochondrial cristae & respiratory chain**

**Loss of cristae organization**

**ATP depletion**

**Oxidative stress**

**Inhibition of complex V**

**Normal cristae architecture**

**ATP synthesis**

**Redox balance**

**Normal complex V function**

**B**


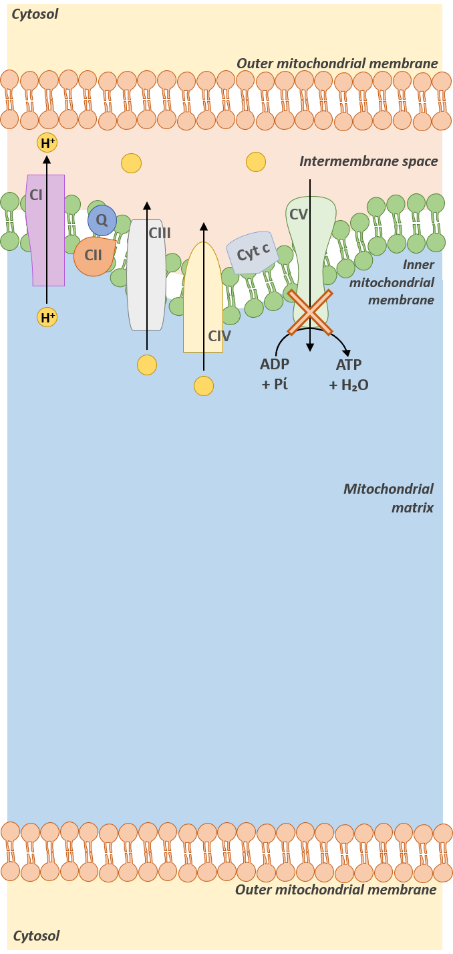


**Mitochondrial cristae & respiratory chain**

**A**

Supplementary Figure 6. **Schematic of cellular events driving TDF toxicity in the kidney. (A) Proximal tubule under normal conditions.** Filtered solutes are reabsorbed across the proximal tubule (PT) apical membrane – either directly or indirectly – with sodium. The basolateral sodium-potassium pumps (Na^+^/K^+^-ATPase) provide the driving force for solute transport, using ATP generated by the respiratory chain (RC). Dimers of complex V (CV) (the ATP synthase) play an important role in maintaining the characteristic folded architecture of the mitochondrial crista, which in turn is crucial for ensuring the optimal positioning of RC complexes (CI – CIV) to maximize the efficiency of oxidative phosphorylation. **(B)** **Proximal tubule in TDF toxicity.** Tenofovir (TFV), the active metabolite of TDF, enters PT cells from the plasma via basolateral organic anion transporters (OAT) and is converted to Tenofovir diphosphate (TFVpp), an inhibitor of complex V. Loss of normal complex V activity leads to: (1) ATP depletion and breakdown of solute transport; (2) abnormal cristae formation and the appearance of enlarged, dysmorphic mitochondria; (3) increasing oxidative stress. Together, these changes explain the mitochondrial phenotype of TDF toxicity in humans.

## Supplementary Tables

| **Patient** | **Age** | **Sex** | **Ethnicity** | **CD4 (cells/ µL)** | **Nadir CD4 (cells/µL)** | **Viral load (copies/ml)** | **eGFR (ml/min/1.73m^2^)** | **ART at biopsy** | **Duration on TDF (months)** | **Duration of HIV infection (months)** | **Renal pathology** | **Potential pathogenic factors** |
| --- | --- | --- | --- | --- | --- | --- | --- | --- | --- | --- | --- | --- |
| 1 | 50 | M | White | 294 | 294 | 50 | 5 | TDF, FTC, ATV/r | 18 | 19 | Acute tubular injury | TDF, T2D |
| 2 | 59 | M | White | 214 | 214 | 50 | 9 | TDF, FTC, ATV/r | 69 | 176 | Acute tubular injury + Diabetic nephropathy | TDF, T2D, ACEi |
| 3 | 43 | M | White | 510 | - | 40 | 6 | MVC, DRV/r | 37 | 218 | Acute tubular injury | TDF, T2D, NSAID |
| 4 | 31 | M | White | 480 | 180 | 50 | 49 | ABC, 3TC, LPV/r | 27 | 31 | Acute tubular injury | TDF |
| 5 | 54 | M | White | 240 | 140 | 50 | 88 | TDF, FTC, LPV/r | 29 | 114 | Acute tubular injury | TDF |
| 6 | 42 | M | White | 1017 | 30 | 50 | 49 | TDF, FTC, LPV/r | 44 | 129 | Acute tubular injury (+IgA on EM) | TDF |

Supplementary Table 1. **Human study cohort characteristics.** M = male; TDF = tenofovir disoproxil fumarate; FTC = emtricitabine; ATV = atazanavir; r = ritonavir boosted; MVC = maraviroc; DRV = darunavir; ABC = abacavir; LPV = lopinavir; T2D = type 2 diabetes mellitus; NSAID = non-steroidal anti-inflammatory drug; ACEi = angiotensin converting enzyme inhibitor; EM = electron microscopy.

| **Quantifying the relative abundance of mitochondrial mtDNA (mtDNA) to nuclear DNA (nDNA)** | | |
| --- | --- | --- |
| **Gene** | **Fwd (**5’--3’) | **Rev (**5’--3’) |
| MT-ND1 | ACACTAGCAGAGACCAACCG | GAAGAATAGGGCGAAGGGGC |
| MT-COX1 (A) | CTAGCAGGTGTCTCCTCTATCT | GAGAAGTAGGACTGCTGTGATTAG |
| MT-COX1 (B) | TTCGCCGACCGTTGACTATTCTCT | AAGATTATTACAAATGCATGGGC |
| ACTB | TCACCCACACTGTGCCCATCTACGA | CAGCGGAACCGCTCATTGCCAATGG |

**B**

**A**

| **PCR conditions** | | | |
| --- | --- | --- | --- |
| **Temperature (°C)** | 95°C | 95°C | 60°C |
| **Time** | 3 min | 40 cycles of 15 sec | 30 sec |

Supplementary Table 2. **Primer sequences and conditions for qPCR experiments. (A)** Gene and **(B)** primer information for measuring the relative abundance of mitochondrial mtDNA (mtDNA) to nuclear DNA (nDNA) in RPTEC/TERT1 cells.

| **Primary/Secondary** | **Antibody** | **Product number** | **Manufacturer** |
| --- | --- | --- | --- |
| Primary | Total OXPHOS WB Antibody Cocktail | ab110413 | Abcam |
|  | MT-ATP6 | A8193 | ABclonal |
|  | α-tubulin | GT114 | GeneTex |
| Secondary | Goat anti-rabbit HRP | 31460 | Pierce |
|  | Goat anti-mouse HRP | 31430 | Pierce |

Supplementary Table 3. **Antibodies used for Western Blot analysis of respiratory chain protein abundance.** HRP = horseradish peroxidase.

**A**(detailed in Supplementary Table 4A)

| **Assay** | **Antibody** | **Concentration** | **Product number** | **Manufacturer** |
| --- | --- | --- | --- | --- |
| Complex I/IV assay | NDUFB8 (CI) | 1:100 | ab110242 | Abcam |
|  | MT-CO1 (CIV) | 1:100 | ab14705 | Abcam |
|  | VDAC1 (OMM) | 1:50 | ab14734 | Abcam |
| Complex III/V assay | UQCRFS1 (CIII) | 1:50 | ab14746 | Abcam |
|  | ATPB (CV) | 1:100 | ab14730 | Abcam |
|  | VDAC1 (OMM) | 1:50 | ab14734 | Abcam |

| **Assay** | **Antibody** | **Concentration** | **Product number** | **Manufacturer** |
| --- | --- | --- | --- | --- |
| Complex I/IV assay | Goat anti-mouse IgG1 biotin | 1:200 | 115-065-205 | Jackson ImmunoResearch |
|  | Goat anti-mouse IgG2b Alexa 488 | 1:200 | A-21131 | ThermoFisher |
|  | Goat anti-mouse IgG2a Alexa 546 | 1:200 | A-21143 | ThermoFisher |
| Complex III/V assay | Goat anti-mouse IgG2b Alexa 647 | 1:200 | A-21121 | ThermoFisher |
|  | Goat anti-mouse IgG1 Alexa 488 | 1:200 | A-21242 | ThermoFisher |
|  | Goat anti-mouse IgG2b Alexa 546 | 1:200 | A-21143 | ThermoFisher |

**B**(detailed in Supplementary Table 4A)

Supplementary Table 4. **Antibody cocktails used for multiplex immunofluorescence staining for respiratory chain complex expression.** (A) Primary and (B) secondary antibody cocktails. OMM = outer mitochondrial membrane, CI = complex I, CIV = complex IV, CIII = complex III, CV = complex V.

## Supplementary Methods

### *Supplementary Methods 1.* ***Preserving dome formation***

In order to preserve dome formation – and to maintain the confluent cell monolayer – culture medium was exchanged using sequential washes. This decreases the volume of old medium whilst increasing the volume of new medium. If the culture medium is fully removed, cells forming domes (which, consequently, are not attached to the plastic plate) are vulnerable to becoming detached from surrounding cells and lost. Exchanging the medium in this way ensures that cells are always submerged.

### *Supplementary Methods 2.* ***Quantifying drug-induced changes in PT cell function in vitro***

### *Supplementary Methods 2A.* ***Immunofluorescence antibody staining***

A rabbit polyclonal (1:200, sc-11415, Santa Cruz) or mouse monoclonal (1:200, sc-17764, Santa Cruz) antibody was used to target the outer mitochondrial membrane protein TOM20. Cells incubated with the latter TOM20 antibody where co-stained with a rabbit polyclonal antibody for LC3 (1:500, PM036, MBL), which is a marker for autophagosomes. Cells were stained with the appropriate secondary antibodies (1:500, 711-546-152, AF488 donkey anti-rabbit, Jackson ImmunoResearch and/or 1:500, a-11004, AF568 goat anti-mouse, ThermoFisher). Both primary and secondary antibodies were prepared in 1% BSA in PBS.

### *Supplementary Methods 2B.* ***Screening drug treatments using high-throughput fluorescence microscopy***

Widefield fluorescence microscopy was performed using a fully automated high-content imaging system (IN Cell Analyzer 2500 HS, GE). Nuclei were imaged using a 10x air objective. The entire area of each cell monolayer was imaged using 25 fields arranged with a 10% overlap. The full volume of the nuclei was collected by acquiring image z-stacks in steps of 5 µm. Mitochondria and autophagosomes were imaged using a 40x air objective. In order to capture a comprehensive overview of the cell monolayers, 30 imaging fields were arranged with fixed spacing in each well. Image z-stacks were acquired with a z-step size of 0.38 µm.

*Supplementary Methods 2C.* ***Image analysis: quantifying cell number and transport function***

Extended Depth of Field (EDF) is an ImageJ plugin which recognizes in-focus information at a specimen’s surface from a stack of images. This allows a 3D structure, such as a monolayer of cells, to be represented as a single in-focus 2D image. This is achieved by merging a z-stack of images taken at numerous focal positions into: 1) an in-focus composite image and 2) a topography height-map of in-focus pixel positions.

We processed each z-stack of nuclei images using EDF. Nuclei from differing focal planes were represented in a composite image. In the topography height-map, nuclei located in a lower focal plane are represented as a darker color, whilst nuclei present in domes are positioned in a higher focal plane and are represented as a lighter color

Using ImageJ, we stitched together the composite images to recreate entire cell monolayers. Likewise, height-maps were stitched together to create a 2D representation of the three-dimensional cell monolayer. In order to improve the accuracy of the eventual segmentations, we performed pre-processing steps to enhance the structures of interest. ‘Enhance Local Contrast (CLAHE)’ was used to increase the contrast between nuclei and background, whilst ‘FFT Bandpass Filter’ was used to increase the contrast between domes and background.

Next, machine learning based image analysis software was used to identify, segment and quantify structures of interest from the stitched images of nuclei and domes. Using the composite images, cell number was assessed by counting all the nuclei in an entire cell monolayer. Approximately, 75,000 cells were present in a confluent monolayer. Using the stitched height-maps, transport function was quantified by calculating the total area of the cell monolayer involved in dome formation. Machine learning software (Ilastik version 1.3.2) was trained to classify and segment nuclei and domes. Once segmented, structures were quantified automatically using the ‘Analyze Particles’ tool in ImageJ. The consistency, accuracy and reliability of the machine learning approach was confirmed by carefully inspecting the automated segmentations and quantifications alongside the original images.

*Supplementary Methods 2D.* ***Image analysis: mitochondrial morphology***

Image z-stacks were loaded into ImageJ. 90 fields of view (48 μm x 48 μm) were selected. We enhanced the contrast of the mitochondrial signal against the background using the ‘Unsharp Mask’ tool. Next, the most in-focus slice from the z-stack was selected. The ‘Enhance Local Contrast (CLAHE)’ tool was used to create a two-dimensional projection of the three-dimensional mitochondrial structures; thus, mitochondrial fluorescence signal in slices above and below the selected z-slice were brought into focus in a single image plane.

Mitochondrial morphology was assessed by calculating the mitochondrial footprint (the total area of each field of view occupied by mitochondria) and the number of enlarged mitochondria per field of view. Machine learning based image analysis software (Ilastik) was trained how to classify structures of interest. Firstly, mitochondria were classified and segmentations of the mitochondrial footprint were generated. Secondly, enlarged mitochondria were classified and size thresholds were applied, allowing the identification of dysmorphic mitochondria against regular shaped mitochondria. Once segmented, structures were quantified automatically using the ‘Analyze Particles’ tool in ImageJ. The consistency, accuracy and reliability of the segmentations was confirmed across a range of images.

*Supplementary Methods 2E.* ***Image analysis: autophagosome formation***

Large autophagosome formation was used as an indicator of cellular metabolic stress. 10 fields of view (331.5 μm x 331.5 μm) were selected. The most in-focus slice from the z-stack was identified, from which large autophagosomes were counted manually. The number of large autophagosomes was normalized to the number of nuclei in the field of view.
